# Observations on the Early Establishment of Foliar Endophytic Fungi in Leaf Discs and Living Leaves of a Model Woody Angiosperm, *Populus trichocarpa* (Salicaceae)

**DOI:** 10.3390/jof4020058

**Published:** 2018-05-16

**Authors:** Yu-Ling Huang, Naupaka B. Zimmerman, A. Elizabeth Arnold

**Affiliations:** 1 School of Plant Sciences, The University of Arizona, 1140 E. South Campus Drive, Tucson, AZ 85721, USA; nzimmerman@usfca.edu (N.B.Z.); arnold@ag.arizona.edu (A.E.A.); 2 National Museum of Natural Science, 1 Guancian Rd., Taichung 40453, Taiwan; 3 Department of Biology, University of San Francisco, Harney 219C, 2130 Fulton Street, San Francisco, CA 94117, USA; 4 Department of Ecology and Evolutionary Biology, The University of Arizona, 1041 E. Lowell St., Tucson, AZ 85721, USA

**Keywords:** *Cladosporium*, colonization, microscopy, *Penicillium*, *Populus*, stomata, *Trichoderma*

## Abstract

Fungal endophytes are diverse and widespread symbionts that occur in the living tissues of all lineages of plants without causing evidence of disease. Culture-based and culture-free studies indicate that they often are abundant in the leaves of woody angiosperms, but only a few studies have visualized endophytic fungi in leaf tissues, and the process through which most endophytes colonize leaves has not been studied thoroughly. We inoculated leaf discs and the living leaves of a model woody angiosperm, *Populus trichocarpa*, which has endophytes that represent three distantly-related genera (*Cladosporium*, *Penicillium*, and *Trichoderma*). We used scanning electron microscopy and light microscopy to evaluate the timeline and processes by which they colonize leaf tissue. Under laboratory conditions with high humidity, conidia germinated on leaf discs to yield hyphae that grew epiphytically and incidentally entered stomata, but did not grow in a directed fashion toward stomatal openings. No cuticular penetration was observed. The endophytes readily colonized the interiors of leaf discs that were detached from living leaves, and could be visualized within discs with light microscopy. Although they were difficult to visualize within the interior of living leaves following in vivo inoculations, standard methods for isolating foliar endophytes confirmed their presence.

## 1. Introduction

Plants in natural and agricultural ecosystems worldwide harbor fungal endophytes in their symptomless tissues, including apparently healthy leaves [1,2]. These endophytes often influence aspects of leaf physiology, gene expression, and function [3,4,5]. Their prevalence and importance prompt inquiries into the processes by which endophytic symbioses establish in living foliage. Despite their occurrence in all of the groups of land plants that have been examined to date [6], little is known regarding the early phases of endophyte colonization in leaves.

Most foliar endophytes are horizontally transmitted and form highly localized infections in leaf tissue (i.e., class 3 endophytes in [6]; hereafter, endophytes). These endophytes have been found in all of the plant species that have been surveyed thus far, and are highly diverse in wild and agroecosystems [6,7,8,9,10,11]. They typically colonize newly flushed leaves as spores or hyphal fragments [2]. After landing on leaf surfaces, most endophytes are thought to enter leaf tissues through stomata or wounds, or in some cases may enter directly through the cuticle [6,12]. Stone [13] reported that certain endophytes in the family Rhytismataceae, which occur in the leaves of Douglas fir, are limited mainly to a single epidermal cell until leaf senescence (ca. two to five years). At that time, formerly quiescent hyphae began to colonize surrounding tissue. Other studies have reported that endophytes occupy intercellular spaces near parenchyma cells [14], or grow slowly between epidermal and hypodermal cells instead of remaining quiescent [15]. These studies, while illuminating, have focused on the foliage of conifers, leaving aside the most diverse and prevalent clade of plants that host horizontally-transmitted endophytic fungi: the angiosperms.

Arnold and Herre [16] and Arnold et al. [17] demonstrated that the endophyte-free leaves of a woody angiosperm can be produced by planting surface-sterilized seeds in sterilized soil and protecting emergent leaves from surface wetting. Plants then can be inoculated by endophytes, providing a basis to study early interactions between endophytes and leaves, the processes of colonization, and—depending on the inoculum—competition, antibiosis, or other traits of the mixed endophyte communities in plant tissues [18].

The aim of this study was to examine how foliar endophytes that were isolated originally from a woody angiosperm establish endophytically within leaf tissues. We focused on inoculation experiments with *Populus trichocarpa* (Salicaceae), which is a model woody plant that can be cultivated readily from cuttings under greenhouse conditions. We examined three endophyte strains that represent three classes of Pezizomycotina (Dothideomycetes, Eurotiomycetes, and Sordariomycetes).

## 2. Materials and Methods

**Figure 1:**
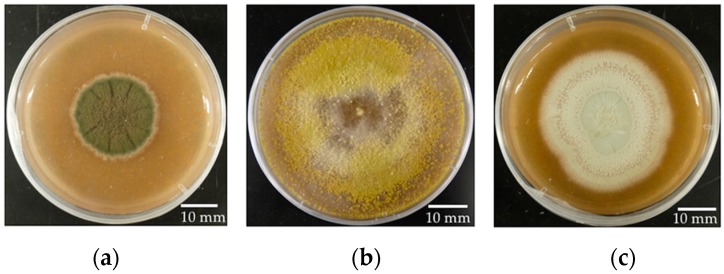
Selected strains of endophytic fungi. (**a**) *Cladosporium tenuissimum* SK2012; (**b**) *Trichoderma* sp. NM2012; (**c**) *Penicillium citrinum* 0079.

We selected three fast-growing fungal strains that were originally isolated from the leaves of *P. trichocarpa*. *Cladosporium tenuissimum* SK2012 and *Trichoderma* sp. NM2012 are endophytes that have been isolated from wild *P. trichocarpa* in Washington State [19]. They are of particular interest because they appear to reduce the severity of plant disease in *P. trichocarpa* [19]. *Penicillium citrinum* 0079 was isolated by Naupaka B. Zimmerman from *P. trichocarpa* leaves collected near Flathead Lake, Montana by Posy E. Busby. The three fungal strains readily produce conidia on V8 media within approximately seven days when incubated at room temperature (Figure 1).

Cuttings of *P. trichocarpa* Nisqually-1 were provided by Steven Strauss (Department of Forest Ecosystems and Society, Oregon State University) from a common garden on the campus of Oregon State University. They were rooted as leafless cuttings in two-gallon pots of Sunshine Mix #4 without mycorrhizae, and grown in a greenhouse at the University of Arizona in Tucson, Arizona. Aside from experimental inoculations as described below, care was taken to avoid the surface-wetting of aboveground plant tissues throughout the experiment to avoid contamination by ambient endophytic fungi [16,17]. Plants were surface-irrigated with a solution of Peter’s Excel 15-5-15 Cal-Mag special fertilizer (Everris NA Inc., Dublin, CA, USA). The temperature in the greenhouse ranged from 7 °C to 35 °C, and humidity ranged from 20% to 90%. All of the plants had produced at least 10 leaves before the start of the experiment.

### 2.1. Leaf Disc Assays

We first conducted an in vitro inoculation experiment to characterize the timeline of endophyte colonization and establishment in leaf tissue. We used leaf discs that were detached from living leaves. Leaf discs were evaluated using light microscopy (LM) and scanning electron microscopy (SEM), as described below (for details regarding microscopy, see Section 2.3 and Section 2.4).

Three leaves from leaf plastochron index (LPI) five to seven were collected from each of six plants on the same day. Each leaf was placed in a Ziploc bag immediately after collection and stored at 4 °C. Within 48 h of collection, leaf discs were cut from healthy leaves with sterile cork borers of the desired size, with large discs (1.2 cm in diameter) and small discs (6 mm in diameter) cut from each leaf for LM and SEM, respectively. Discs were washed by vortexing for one minute in sterile 0.1% Tween 20, and rinsed in double autoclaved water. Five 1% water agar plates in 150-mm Petri dishes were prepared for each leaf. Three plates received nine large discs each, and one plate received 24 small discs. One plate received six large discs as a control. Leaf discs were placed so that the abaxial epidermis was facing up. Agar plates with leaf discs were sealed with Parafilm and stored at 4 °C for up to two days before inoculation. Leaf discs were green and turgid at inoculation time.

After comparing fungal growth and conidia production on several types of common fungal media, we found that potato dextrose agar amended with 1% yeast extract (PDA + 1% YE) induced the most abundant conidia production across our three focal strains. Inoculum was prepared by culturing each fungal strain on PDA + 1% YE for 16 days at approximately 21 °C. Five mL of sterile 0.1% (*v/v*) Tween 20 was added to each plate, and conidia were gently dislodged with a sterile rubber policeman. The concentration of conidia was quantified with a hemocytometer and adjusted to 10^6^ conidia/mL [17]. A total of 30 µL and 5 µL of conidia solution was inoculated onto each large and small leaf disc, respectively. Plates were sealed by Parafilm and incubated in room temperature (ca. 21 °C) with approximately 12-h light/dark cycles.

We expected the initial stages of spore germination to be mainly on the leaf surface, which we evaluated by SEM within three days. Later entry was evaluated by LM for up to 21 days. After three days, samples were no longer of high enough quality for SEM, as water loss led to tissue shrinkage, which interfered with sample visualization.

For SEM, three small discs were collected at 0 h, 6 h, 12 h, 24 h, 48 h, and 72 h after inoculation and placed in 1% glutaraldehyde in 0.1-M phosphate buffer. For LM, three large discs were collected at 0 days, 1 day, 2 days, 3 days, 5 days, 7 days, 10 days, 14 days, and 21 days after inoculation, immersed in 1 M of sterile potassium hydroxide (KOH), and stored at room temperature. Samples for time point 0 for both LM and SEM were collected immediately following inoculation. We had two types of negative controls: three discs were inoculated with the standard amount of sterile 0.1% Tween 20 (but no conidia), and three discs were prepared without any inoculation solution. Both types of controls were collected at 72 h and 14 days after inoculation for SEM and LM, respectively (see below).

### 2.2. In Planta Assays

Since leaf discs are detached from living leaves, leaf disc assays may provide only partial insight into how endophytes interact with living tissue. Therefore, we introduced endophytes into the leaves of living plants under greenhouse conditions.

Sixteen plants were selected randomly for in vivo inoculation. The inoculum was prepared as described above, except that conidial suspensions were transferred to sterile hand-held spray bottles. Prior to use, bottles were soaked in dilute commercial bleach (0.5% sodium hypochlorite) for 10 min, and then rinsed multiple times with autoclaved water. The viability of conidia in each inoculation solution was evaluated by spraying three times into a 50-mL sterile Falcon tube (mimicking foliar application), and then pouring the solution onto a plate with V8 agar. Fungal growth on the plate was confirmed by light microscopy three days later.

For each plant, seven leaves that ranged from LPI 5 to 11 were inoculated concurrently. The LPI of each leaf at the time of inoculation was recorded as the original LPI for later reference. Each leaf was covered with a plastic produce bag and inoculated by spraying the specified conidial suspension onto both leaf surfaces. Each bag was then sealed around the petiole to maintain high local humidity for 36 h. One leaf per plant was collected at 0 days, 1 day, 2 days, 3 days, 4 days, 7 days, and 14 days after inoculation. Leaves collected on day 0 were collected immediately after inoculation, and leaves collected on day 0 and 1 were collected before the plastic bags were removed. Leaves were collected in descending order of the original LPI, from LPI 11 to 5 (i.e., a leaf that was originally designated as LPI 11 was collected on day 0, a leaf that was originally designated as LPI 10 was collected on day 1, and so on). Each leaf was placed in a Ziploc bag and carried in a cooler to the lab for processing.

Leaves were processed immediately after collection. At each time point, 10 discs (6 mm in diameter) were cut from each leaf for SEM. An additional 10 pieces of leaf tissue (ca. 0.5 cm^2^) were cut from each leaf for sectioning by microtome. Leaf tissues for sectioning were placed in FAA (formalin:acetic acid:ethanol = 5:5:90), vacuumed to remove air bubbles, and stored at 4 °C.

For samples collected 0 and 14 days after inoculation, half of each leaf piece was processed for SEM and microtome sectioning immediately after collection. The other half was put in a Ziploc bag and stored at 4 °C overnight for culturing.

We used a culturing approach to determine whether inoculation was successful per traditional methods of endophyte isolation from leaf tissue. Thirty discs (6 mm in diameter) were cut from leaves collected on day 0 and day 14. Discs were washed by vortexing first in sterile 0.1% *v/v* Tween 20 for 30 s, and then in autoclaved water for 30 s to remove the fungi that was present on the leaf surface. The washed discs then were placed on V8 agar amended with CaCO_3_ [20]. Fungi that emerged from the leaf discs were examined and identified based on morphology. For those fungi that could not be identified conclusively by morphology, genomic DNA was extracted, and the nuclear ribosomal internal transcribed spacers (ITS) and 5.8S gene, and an adjacent portion of the nuclear ribosomal large subunit (partial LSU) were amplified as a single fragment by PCR and sequenced following U’Ren et al. [21].

### 2.3. Sample Preparation for Light Microscopy

We used 1 M of KOH to clear leaf discs and remove cellular materials so that the endophytes could be visualized with LM on and within leaf tissues without interference by leaf pigments (modified from [13]). The KOH was changed several times until the solution and leaf discs became clear. Usually, the leaf discs were clear enough for microscopy in one month, but the time varied depending on leaf toughness and coloration.

Once the leaf discs were clear, they were transferred gently to a 35-mm Petri dish and rinsed three times with distilled water for 10 min each; then, they were stained by 0.05% trypan blue in lactoglycerol (lactic acid:glycerol:water = 1:1:1) for 2 h (modified from [22]). Discs then were destained in lactoglycerol for at least 30 min before examination by LM.

### 2.4. Sample Preparation for SEM

We used SEM to examine spore germination, the growth on leaf surfaces, and entry points into leaves by endophytes. Leaf discs stored in 1% glutaraldehyde were transferred to half-strength Karnovsky’s fixative (Electron Microscopy Sciences, Hatfield, PA, USA) for 4 h and rinsed with 0.1 M of phosphate buffer three times for 20 min each, followed by an ethanol series dehydration (in sequence: 30%, 50%, 70%, 80%, 90%, 95%, and 100%; modified from [23]). Each dehydration step was 25 min. Discs then were immersed in 100% ethanol overnight before critical point drying using a Polaron Critical Point Drier (Polaron, Hertfordshire, UK). Leaf discs were mounted on SEM stubs using carbon conductive tabs and then coated with platinum using a Hummer 6 Sputtering coater (Anatech, CA, USA, Hayward, CA, USA). Sample stubs were examined by Hitachi S-4800 Field-Emission (Hitachi, Tarrytown, NY, USA) SEM at the University of Arizona’s University Spectroscopy and Imaging Facilities (USIF). We quickly examined each leaf disc under low magnification and then took several photos at random with a magnification of 300× once patches of conidia or fungal growth were found. At this magnification, the area represented in each photo was approximately 422 µm × 276 µm. The number of conidia per photo was counted for reference.

### 2.5. Microtome Sectioning

We used microtome sectioning to obtain microsections of leaves from the in vivo inoculation experiment. From observations with SEM, we found that leaf discs inoculated with *Cladosporium* and *Penicillium* had fungal growth at 14 days after inoculation; as a result, we focused on these two fungi for the sectioning work. In particular, we selected samples from leaves with the highest reisolation frequency in the culturing test.

Prior to sectioning, we used Technovit 7100 (Electron Microscopy Sciences, Hatfield, PA, USA) as the embedding medium. The sample preparation protocol followed Tobe and Kadokawa [24]. Briefly, leaf tissue stored in FAA was transferred to 50% ethanol with a few drops of 1% safranin O and incubated for 4 h, followed by ethanol series dehydration (70%, 80%, 90%, 95%, and 100%). Each dehydration step was 4 h to 12 h, depending on the tissue size and thickness, and the final step was repeated to ensure complete dehydration. The plastic embedding medium was compatible with 100% ethanol, and five pre-embedding solutions in different concentrations of Technovit 7100 were prepared for filtration. Samples were immersed in each filtration solution for 12 h to 24 h; then, each piece of tissue was transferred to each block of HistoForm S for embedding and polymerization at 4 °C overnight. The ratio of the embedding media Technovit 7100: hardner II: polyethylene glycol 400 (PEG400) was 15:1:0.6. Adding PEG400 can soften the embedding medium, which facilitates the cutting by disposable microtome knife [25]. Samples were suspended in blocks with Histobloc and Technovit 3040. Blocks were removed from the block mold, trimmed, and re-embedded for proper orientation before cutting by microtome. Microsections at a thickness of six µm were cut by an American Optical Spencer No. 815 (American Optical, Buffalo, NY, USA) with Tissue-Tek high-profile disposable microtome blades (Sakura Finetek USA Inc., Torrance, CA, USA). Five blocks were cut to represent each fungal strain. Forty microsections were aligned in order on one glass slide, and five slides were made for each embedded block. Slides were stained by cotton blue and safranin O [26].

## 3. Results

### 3.1. Leaf Disc Assays

Leaf discs inoculated in the in vitro experiment were robust to clearing by KOH prior to staining and LM, and the methods described above were also appropriate for fixing and preparing samples for SEM. Overall, we found that conidia were unevenly distributed near the central area of each leaf disc when examined by SEM. In general, conidia were visible at each time point. Most of the stomata were closed in the SEM samples, but most were open in the LM samples. The time and frequency of conidial germination varied among fungal strains, as described below.

**Figure 2:**
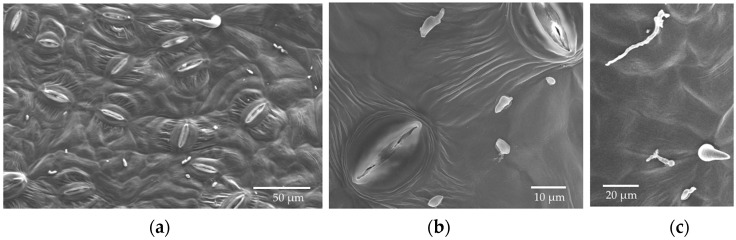
SEM photos from the in vitro inoculation of leaf discs of *P. trichocarpa* with an endophytic strain of *Cladosporium tenuissimum* SK2012 (**a**) Low magnification view of lower epidermis, six hours after inoculation (HAI); (**b**) Germinated and non-germinated conidia, 12 HAI; (**c**) Germinated conidia, 24 HAI.

**Figure 3:**
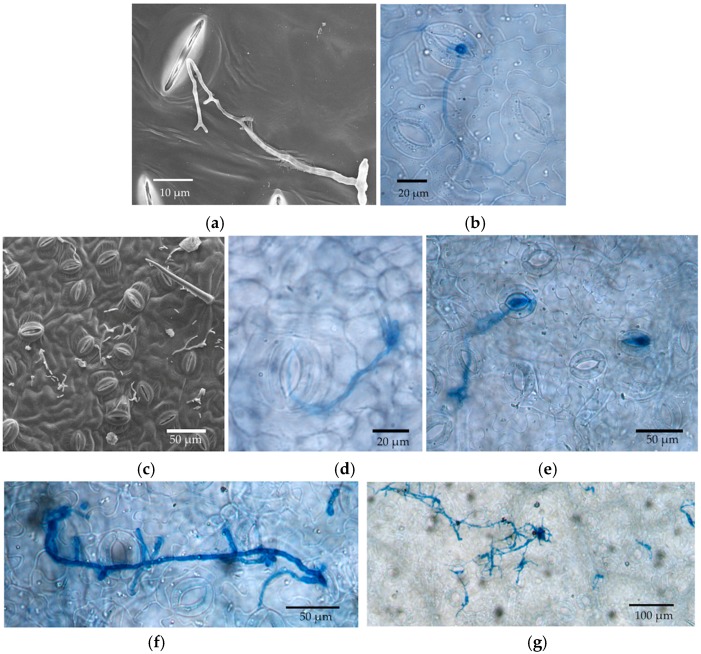
Photographs of hyphal growth from the in vitro inoculation of leaf discs of *P. trichocarpa* with *Cladosporium tenuissimum* SK2012. (**a**) Scanning electron microscopy (SEM), two days after inoculation (DAI); (**b**) Light microscopy (LM), 2 DAI; (**c**) SEM, 3 DAI; (**d**) LM, 3 DAI; (**e**) LM, 7 DAI. (**f**) LM, 10 DAI; (**g**) LM, 14 DAI.

**Figure 4:**
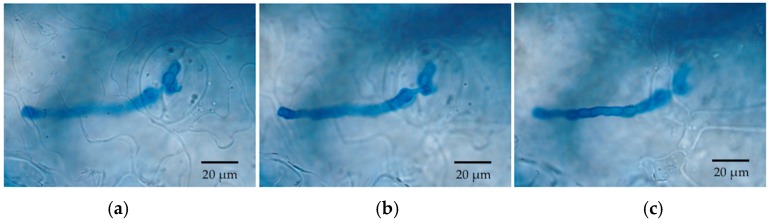
*Cladosporium* hypha entering a stoma of a leaf disc of *P. trichocarpa*. (**a**) Focused on the leaf epidermis; (**b**) Focused right below epidermis; (**c**) Focused within deeper leaf tissue.

Germinated conidia of *Cladosporium* were observed by SEM at 6 h after inoculation (HAI) (Figure 2). A small number of germinated conidia were observed at 12 HAI (Figure 2), and most conidia had germinated by 24 HAI (mean, 91%; Figure 2). Germinated conidia also were observed by LM. Extensive growth of fungal hyphae could be seen on leaf discs at two days after inoculation (DAI; Figure 3). SEM showed that fungal hyphae grew readily on the leaf surface, with growth apparently random rather than directed toward stomatal openings. However, we did not see direct evidence of hyphae entering plant tissues with SEM, and we found that some hyphae were rejected by stomata or turned away from them (Figure 3). However, examination by LM showed that conidia were usually in or close to the stomata (Figure 2 and Figure 3), and some hyphae were entering and growing inside leaf discs (Figure 4).

**Figure 5:**
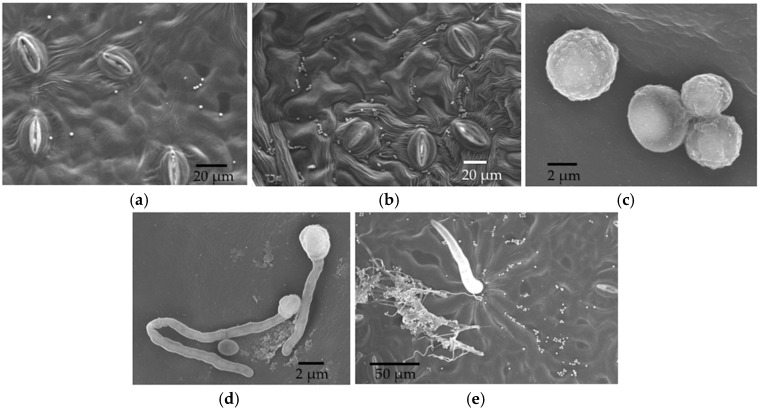
SEM photos from the in vitro inoculation of leaf discs of *P. trichocarpa* with an endophytic strain of *Penicillium citrinum* 0079. (**a**) Low magnification view of lower epidermis, six HAI; (**b**) Non-germinated conidia are dispersed on lower epidermis, 24 HAI; (**c**) Non-germinated conidia at high magnification, 24 HAI; (**d**) Germinated and non-germinated conidia at high magnification, 48 HAI; (**e**) Germinated conidia and hyphae, as well as non-germinated conidia, on lower epidermis, 48 HAI.

**Figure 6:**
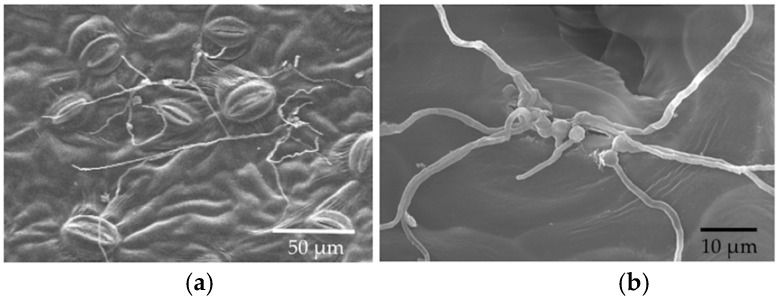

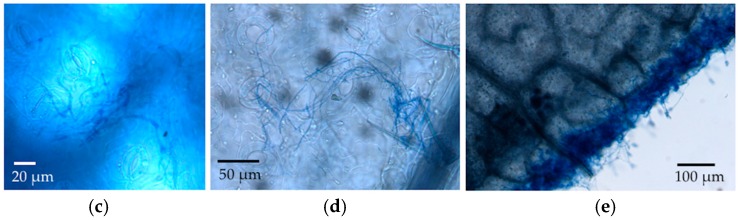
Hyphal growth on leaf discs of in vitro inoculation with *Penicillium citrinum* 0079. (**a**) SEM at low magnification, 3 DAI; (**b**) SEM at high magnification, 3 DAI; (**c**) LM, 7 DAI; (**d**) LM, 14 DAI; (**e**) LM at the margin of leaf disc, 3 DAI.

No germinated conidia of Penicillium were found on leaf discs at 6, 12 and 24 HAI (Figure 5), but germinated conidia were observed by SEM at 48 HAI (Figure 5). The germination rate was less than 50% overall (Figure 5). Extensive growth of hyphae could be seen by SEM and LEM at 48 HAI or later (Figure 6). Conidia that germinated around stomata grew hyphae in random directions (Figure 6). Examination by LM showed that the margins of leaf discs were colonized extensively by fungal hyphae by 3 DAI, and some were producing conidia (Figure 6). Extensive hyphal growth across leaf surfaces was observed by LM at 3 DAI (Figure 6), but we were unable to distinguish the hyphae from inoculation/spore germination vs. growth from the disc margins.

**Figure 7:**
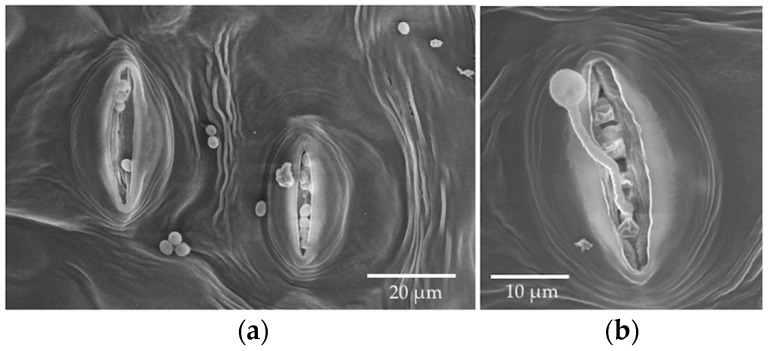
SEM photos from the in vitro inoculation of leaf discs of *P. trichocarpa* with an endophytic strain of *Trichoderma* sp. NM2012 at 72 HAI. (**a**) Non-germinated conidia; (**b**) A germinated conidium with hyphal penetration into the stomatal opening.

**Figure 8:**
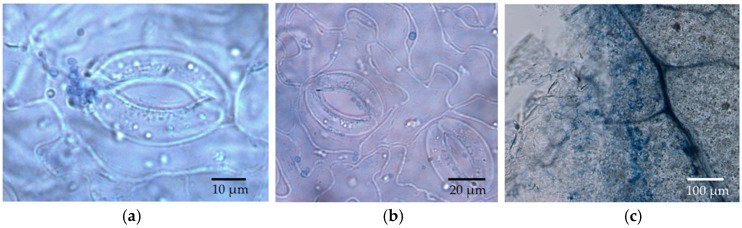

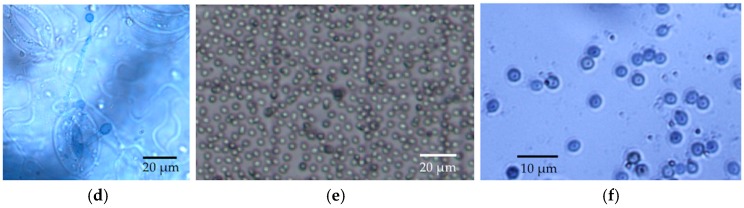
LM photos of in vitro inoculation with *Trichoderma* sp. NM2012. (**a**) Non-germinated conidia, 7 DAI; (**b**) Non-germinated conidia, 14 DAI; (**c**) Hyphal growth at the margin of leaf disc, 3 DAI; (**d**) Chlamydospores, 3 DAI; (**e**) Conidia from fresh cultures on hemocytometer; (**f**) Chlamydospores from a four-month culture.

For *Trichoderma*, we examined SEM samples only at 72 HAI, as very few geminated conidia were found before this time (Figure 7). We observed hyphae from germinated conidia entering stomatal openings (Figure 7). LM at seven and 14 DAI showed a few non-germinated conidia near center of each leaf disc (Figure 8), but extensive fungal growth with conidia and chlamydospores were found at the margin of leaf discs as early as three DAI (Figure 8). We could not distinguish inoculated conidia from the conidia produced from the leaf margin. In addition, chlamydospores were produced at the leaf margin and dispersed on the leaf disc by three DAI (Figure 8). Conidia from the freshly prepared cultures were small and did not have thick walls compared with chlamydospores produced in older cultures (Figure 8).

### 3.2. In Planta Assays

**Figure 9:**
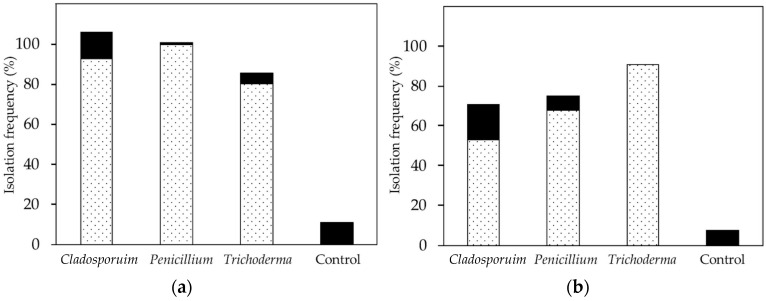
Results of culturing after in vivo inoculation. Dotted: reisolated fungi from inoculation trials, black: strains apparently present in the greenhouse. (**a**) Day of inoculation (i.e., day 0), isolation frequency higher than 100% because some discs yielded more than one isolate; (**b**) 14 DAI.

Overall, 80–100% of the leaf discs that were collected immediately after leaf inoculation in vivo (i.e., on day 0) yielded fungal growth in culture. Non-inoculated fungi were present on 1% to 13% of leaf discs per set (Figure 9). Overall, 53% to 91% of leaf discs collected at 14 DAI yielded fungal growth in culture. At that time, non-inoculated fungi were present on 17% of discs (Figure 9). Controls consistently lacked the strains that were used in the inoculation trials, and fungi were isolated rarely from control leaves.

**Figure 10:**
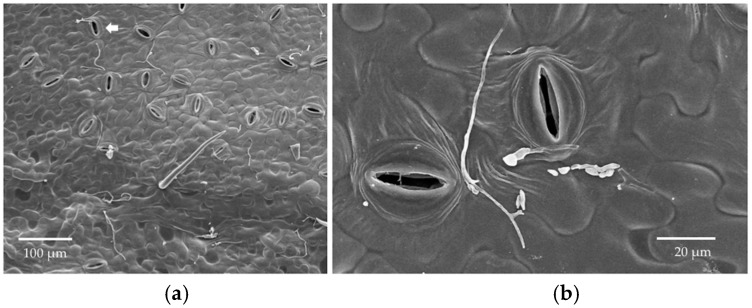

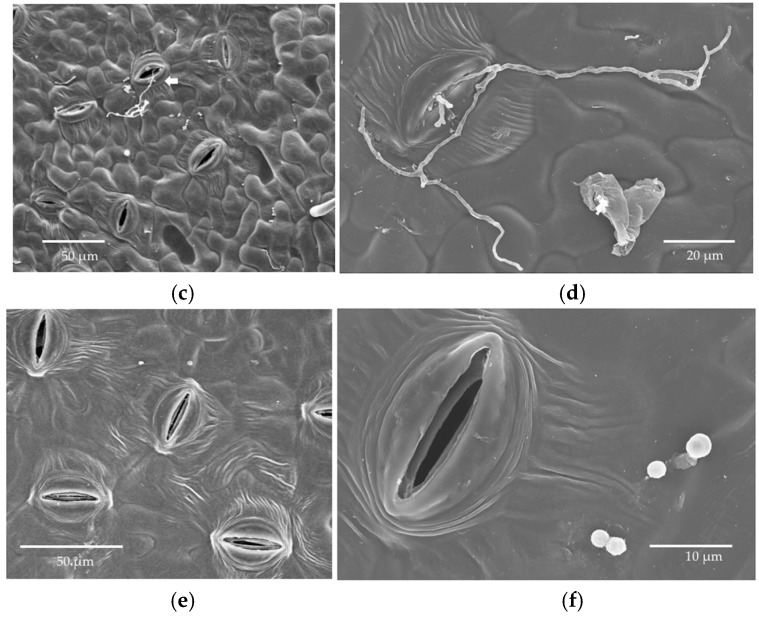
SEM photos of samples from in planta inoculations, 14 DAI. White arrow: hyphal entry through stomata. (**a**,**b**) *Cladosporium*; (**c**,**d**) *Penicillium*; (**e**,**f**) *Trichoderma*.

At 14 DAI, three leaf discs per leaf were examined by SEM. The greenhouse was not a sterile environment, so the leaves were not as clean as the leaf discs that were used in the in vitro inoculation. However, few bacterial cells and fungal spores were found on the control leaves, which was consistent with the culturing results presented in Figure 9. Unlike the stomata that were on the discs examined in the leaf disc assay, some stomata were open (Figure 10).

**Figure 11:**
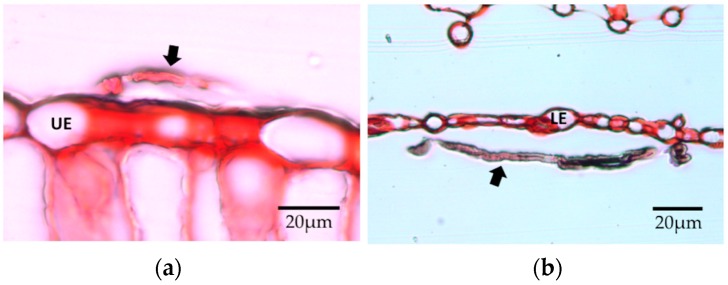
Sections of leaves from in planta inoculations, 14 DAI. Arrow: fungal hypha, UE: upper epidermis, LE: lower epidermis. (**a**) *Cladosporium*; (**b**) *Penicillium*.

Germinated conidia and the extensive hyphal growth of *Cladosporium* and *Penicillium* were observed on the surfaces of inoculated leaves, and non-germinated conidia also were found around them (Figure 10). We did not see evidence that the hyphae of these species grew toward stomatal openings in all cases. Some grew on the leaf surface along the furrows between epidermal cells (Figure 10). Several appeared to enter the stomata (Figure 10, arrows). The conidia of *Trichoderma* were rarely found, and none had germinated (Figure 10). Microtome sectioning showed evidence of hyphae of *Cladosporium* on the upper epidermis and *Penicillium* on the lower epidermis (Figure 11), but no fungi were observed within leaf tissues.

## 4. Discussion

The aim of this project was to visualize the timeline and processes that were relevant to the establishment of endophytic symbioses in the leaves of a model plant. By combining leaf disc assays with in vivo inoculations of living leaves, and examining tissues with scanning electron microscopy and light microscopy, we were able to observe conidial germination, early hyphal growth, and entry into stomata by three endophytic fungi. The endophytes studied here differed in the timing and prevalence of conidial germination, grew in an undirected fashion across leaf surfaces when conditions were humid, engaged in saprotrophic growth on leaf discs, entered stomata (but not in high frequency), and were capable of growth within plant tissues. They were particularly difficult to visualize in leaves after in vivo inoculations, but could be isolated with standard methods from leaves that were inoculated in vivo.

The endophytes that were chosen for this work represent three classes of Ascomycota, and thus are phylogenetically diverse. At the same time, they appear to have converged on endophytic lifestyles, at least for part of their life cycles. These strains are different from many endophytes in that they grow rapidly and conidiate readily in culture, providing ample inoculum for these experiments.

The strains of *Cladosporium* and *Penicillium* examined here grew readily on the leaf surface and did so in an undirected manner with respect to stomatal openings. Similar growth has been reported in a previous study of pathogenic *Cladosporium* (*C. fulvum*) [27]. In that study, virulent and avirulent races of *C. fulvum* were evaluated. Neither race displayed hyphal growth toward stomata, but they differed in terms of callose deposition along the hyphae inside leaf tissues (a plant defense response [28]), and the avirulent strains did not sporulate [27]. We observed hyphae bypassing or being rejected by stomata, which is consistent with De Wit’s observations [27]. However, it is possible that hyphal growth might be more directed under different conditions (e.g., under drier conditions, when stomatal openings might be targeted).

We observed via SEM that most stomata were closed in the leaf disc experiment, but frequently they were open in material collected from the in vivo experiment. Humidity, light, temperature, physiological shock, and chemical agents can alter the movement of guard cells around stomata [29,30], and the closing of stomata is a defensive response to the presence of some microbes [31,32]. The accumulation of potassium in guard cells is critical in controlling stomatal opening [33]. Open stomata in LM samples could reflect abundant potassium ions in the KOH, which was used for clearing leaf discs. These ions can flux into guard cells when the dead plant cells lose active potassium transporters in their cell membranes, thus changing the osmosis and shape of the guard cells to open the stomata. Although the humidity in the sealed agar plates should be higher than in the open air, leaf discs in the in vitro experiment did not have the nutrient and water supplies. Thus, it is possible that the stomata were closed due to this stressful environment, or in response to the inoculated fungi themselves. Whether endophyte hyphae are actively or passively rejected by the stomata remains an interesting avenue for further research.

### 4.1. Methods for Visualization of Fungal Endophytes in Leaves

Clearing and microsections of leaves for LM, SEM, and TEM have been used in the visualization of endophytes from tree leaves [13,14,15], but these have focused on gymnosperms (particularly Pinaceae), which are inhabited by fungi that include endophytic Rhytismataceae with the apparently rare habit of often occurring primarily within single epidermal cells. It is thought that the endophytes of angiosperms generally occur between cells in leaves, which is consistent with *Cladosporium* as depicted in [34]. Our visualization with LM was consistent with the intercellular growth in leaf discs by the endophytes considered here; however, we were not able to observe such growth using the microtome sections of leaves from the in vivo inoculation.

Leaf disc assays have been used extensively in plant pathology [35,36,37,38], but have been seldom applied in endophyte studies. One challenge they present is that leaf discs may encourage a saprotrophic growth phase that is not representative of endophytic growth: leaf discs have limited access not only to water and nutrients, but also to defense, when compared with living leaves. Most control (non-inoculated) leaf discs in our experiment were still green on day 14, but some became slightly discolored. Thus, leaf discs were likely under water stress, and may have had a reduced ability to respond to infection relative to leaves in planta. In general, water stress can cause plant cells to shrink, decrease leaf area, increase the synthesis of abscisic acid (ABA) and ethylene, decrease stomatal conductance, and reduce the rate of photosynthesis. Exploring the relevance of these factors for endophyte colonization is of interest for future work, as it is possible that one or more may have influenced the results of this study.

The three fungal genera used in this study are distributed widely, and are thought to have saprotrophic life phases in addition to their endophytic modes [19,39,40,41]. Saprotrophic stages were observed in the in vitro inoculation, in which we found rapid growth of *Penicillium* and *Trichoderma* at the leaf disc margins. It is possible that the endophytes chosen for this work are biased somewhat toward saprotrophy and are only opportunistic as endophytes, warranting the exploration of other endophytic fungi of angiosperms with different life histories.

### 4.2. Visualizing Colonization in Living Leaves

Previous studies have reisolated endophytes from inoculated plants after 14 days, but report more successful isolations after 28 days or more [17]. We found that after 14 days, we frequently isolated the inoculated strains from living leaves, suggesting that *Cladosporium* and *Penicillium* did establish endophytic associations in vivo. If proliferation within tissues is relatively limited after 14 days, then perhaps a small amount of intercellular mycelium would be missed by our sectioning and visualization approach (Figure 10). Thus, a longer period between inoculation and evaluation may be useful: over time, more intercellular growth might yield more visible infections [17]. A challenge with our study system is that the individual leaves of *P. trichocarpa* were relatively short-lived in the greenhouse conditions used here, precluding long periods of incubation before harvesting. One useful way forward would be to apply these methods to mature leaves of *P. trichocarpa* in the field, wherein culturing studies suggest there exists a relatively high density of natural infections. We could then improve the methods to make them more sensitive and effective for detecting recent inoculations.

Fungal endophytes are highly diverse, even in a single leaf [6,7,42]. The three distantly related strains that were used in this study represent a small part of the vast diversity of endophytes at a global scale, and thus may not be universally representative of plant–endophyte interactions. Additionally, interactions such as competition and priority effects among fungal strains in living leaves are not captured in this report, and within-species variability among particular fungi or plant genotypes was not considered. Future studies could examine a range of isolates within focal clades of interest, seeking phylogenetically conserved modes of infection in diverse hosts. Despite these caveats, the interactions that we documented here contribute three case studies toward understanding foliar colonization by fungal endophytes in the context of woody angiosperms.
